# Swedish doctors’ experiences and personality regarding medical specialty choice: a qualitative study

**DOI:** 10.5116/ijme.5c60.1c63

**Published:** 2019-02-28

**Authors:** Caroline Olsson, Susanne Kalén, Cecilia Mellstrand Navarro, Sari Ponzer

**Affiliations:** 1 Department of Clinical Science and Education, Södersjukhuset, Karolinska Institutet, Stockholm, Sweden

**Keywords:** Specialty choice, qualitative research, content analysis, recruitment, medical doctors

## Abstract

**Objectives:**

To explore an understanding of medical doctors’ entire process of specialty choice with a focus on the influence of personal experiences and personality traits on choices made.

**Methods:**

A qualitative study was performed. Semi-structured individual interviews were conducted with medical doctors undergoing their specialty training in Sweden about their experiences and personalities. The transcribed interviews were analyzed with an inductive content analysis approach.

**Results:**

A total of 15 medical doctors participated. Three themes were identified using content analysis: To be invited or not, to fit in or not and to contribute or not. Furthermore, the results refute that specialty choice is a long-term, complex process.

**Conclusions:**

First, the importance of being invited to the specialty choice was stressed by the doctors, especially in their early years when they needed to feel valued and trusted. Secondly, the need to fit in was essential to make a sustainable career choice. Finally, the doctors’ expressed a will to contribute to the medical field of their chosen specialty. The interviews showed that specialty choice is a long-term, complex process; therefore, one implication for the healthcare sector would be to target the entire chain of medical education to improve recruitment strategies for those specialties with recruitment difficulties. More studies are needed to understand better how positive and negative encounters within the healthcare sector can influence young doctors’ specialty choice.

## Introduction

Difficulties in recruiting and retaining specialist-trained doctors in particular medical specialties, such as primary care, geriatrics, and psychiatry, pose a problem for the whole healthcare sector. This has long been reported and from different parts of the world.^1^^-^^4^ In addition to recruiting a sufficient number of doctors, it is also essential that the doctor has the right personal qualities for the specialty. From a societal perspective, it is important to employ suitable doctors who can contribute to a particular field.^5^ For the individual doctor, there are incentives for making a good choice regarding specialty. First, specialties suit different types of personalities to varying degrees.^6^^, ^^7^ Some doctors are interested in communication, others are good at swift decision making, whereas some prefer working with their hands, etc. Secondly, specialty choice can be crucial to the individual doctor’s likelihood of remaining in the profession for their entire career. Being in the ‘wrong’ specialty can lead to dissatisfaction, stress, and exhaustion. Landon and colleagues^8^ concluded that the doctors’ degree of satisfaction with their career choice varies between different specialties and that dissatisfaction can lead to medical malpractice or a decision to leave medicine through early retirement or cutting down on practice hours.

Earlier research has reported associations between specialty choice and a range of different, single factors, such as the composition of the student population (gender, social background, etc.),^9^ lifestyle factors^10^^,^^11^ and personality traits.^6^ A literature review on specialty choice from 2016^12^ concluded that intersections between different factors must be analyzed to understand the complexity of specialty choices and that this requires a qualitative research approach. The great number of quantitative studies have, to a varying degree, been successful in finding associations between different factors and specialty choices. However, there are some problems with using only a quantitative research approach. Even when researchers investigate how multiple variables work together to explain specialty choice, it does not expound upon the choice itself but instead relates only how common a particular factor or group of factors are in different specialties. Most of these studies assumed that choice is rational and conscious and, therefore, miss the unintentional aspects of the choosing process.^13^^,^^14^ One early attempt to understand the process of choice by using a theoretical framework of choice itself was made in 1997 by Burack and colleagues.^15^ They concluded that there was extensive research about specialty choice determinants, but almost nothing had been written about the process of choice itself, or, in their words: ‘little attention has been paid to how choosers choose’. The aim of this study, therefore, is to gain an understanding of medical doctors’ entire process of specialty choice with a focus on the influence of personal experiences and personality traits on choices made.

## Methods

### Study design

We performed a qualitative interview study, using content analysis^16^ since this approach best suited our intention to understand the entire process of choice. The use of qualitative methods gave us the opportunity to capture those long-term, complex circumstances the study participants thought had a bearing on their specialty choice. In-depth interviews with open questions provided an opportunity for the study participants to bring up various and sometimes contradictory aspects of their choosing processes. We also gained information about how satisfied doctors in specialty training were with their choice of specialty.

The epistemology behind this study was interpretivism,^17^ which involves the notion that knowledge is subjective and constructed. In the interpretivism tradition, the researcher is aware that all results are based on the researchers’ interpretations of the participants’ description of experiences. Knowledge of a phenomenon, in this case, medical doctors’ specialty choices, is constructed between the researcher and the study participants. In line with this epistemology, personal choices are understood in terms of processes and not necessarily regarded as rational.^18^ We used content analysis to interpret the interviews, and that generated a picture of the phenomenon in question.

### Sampling

A purposeful sampling strategy was used to contribute to variation in participants.^16^ To obtain a diverse cohort, the participants were recruited from six different specialty areas: primary care, internal medicine, geriatrics, psychiatry, surgical specialties, and hospital service. The number of specialties was judged to yield sufficiently rich data.^16^ We aimed to include an equal number of men and women. Both sexes (in total seven males, eight females) were represented in all six specialty areas of this study. The participants were between 30–41 years old; the median age was 33.

### Recruitment of participants and setting

Data collection was conducted in 2017 and resulted in 15 individual semi-structured interviews.^19^ The study participants consisted of 15 doctors undergoing specialty training (residency program) in the Stockholm area.

The participants were recruited in two steps. First, emails were sent to the heads of department medical units in Stockholm County that provide and are responsible for specialty training. They were asked to forward the invitation to doctors in specialty training. After that, we contacted the doctors in training personally and invited them to participate in the study. Anonymity was maintained, as the heads of the departments were unaware which individuals had accepted the invitation.

The research was performed by the Helsinki Declaration. The Regional Ethical Review Board in Stockholm concluded that no ethical permission was required according to Swedish law (registration number 2017/699-31/5). Participants received written, and oral information that participation was voluntary, and that consent could be withdrawn at any time, without explanation or consequences. All participants signed a written letter of consent.

### Data collection

#### Interviews

An interview guide was created in a semi-structured way^19^^,^^20^ and used to achieve a rich description of all aspects that mattered in the choice of specialty.^20^ Following two pilot interviews, a few modifications were made.^21^ Each interview began with open questions about the participants’ experiences and thoughts about their specialty choice. Subsequently, more specific questions were posed regarding personality, earlier education, work experiences and the participants’ perceptions of different specialties. All interviews were conducted by the first author (CO). Interviews were audio recorded and transcribed verbatim.^22^ The data consists of 15 hours and 40 minutes of interview time; the longest interview took 100 minutes, and the shortest lasted for 39 minutes. Data collection was performed iteratively and we had a continued discussion throughout the data collection process to evaluate if sufficient data had been obtained. After 15 interviews, the research group considered the data to be sufficient.^20^^, ^^23^

#### Trustworthiness

Small-scale studies lack generalisability, and our findings must be seen in that light.^20^ With that in mind, we believe that we chose to study participants who could contribute to answering the research questions and illuminating the process of choice. We included both men and women and made sure that both sexes were represented in all investigated specialist areas. We also recruited study participants from different kinds of healthcare providers, e.g., two hospitals, two healthcare centers and from six different specialist areas to obtain broad data.^16^ We believe that our efforts contribute to the credibility of this study.^24^

Our triangulation^24^ contributed to fruitful discussions in all stages of the research process, from study design to the final paper. In this process, we in the research group had different pre-understandings of the studied phenomena and could, therefore, add a variety of perspectives to the studied topic. Our group consisted of a professor in orthopedics (SP), a registered nurse with a PhD in medical education research (SK), a PhD and specialist in hand and orthopedic surgery (CMN) and the first author, a PhD student in medical education research with a background in educational sociology (CO). During the research process, we worked in a reflexive way, inspired by the following statement by Guillemin and Gillam: ‘Research is primarily an enterprise of knowledge construction. The researcher (and co researchers), with his or her participants, is engaged in producing knowledge. This is an active process that requires scrutiny, reflection, and interrogation of the data, the researcher, the participants, and the context that they inhabit.’^25^ Methods and proceedings have been described with as much transparency and detail as possible in order for the reader to determine whether our findings are transferable^24^ to other contexts.

### Data analysis

The analysis process was performed with the inductive content analysis approach based on the descriptions by Graneheim and colleagues.^26^^,^^27^ The analysis process started with the first author (CO) reading through all the transcripts to get a sense of the data as a whole. Then the transcripts were transferred into Envivo 11 Pro for Windows for open coding. According to this inductive approach, sometimes referred to as a data-driven approach, the codes were derived from the data and not decided on beforehand.^27^ When the coding was completed, the first author (CO) identified similarities and differences in the coded material and created sub-categories that were clustered into main categories. The main categories laid the foundation for the creation of three themes, as illustrated in Table 1. Both manifest and latent content were analyzed, the former referring to what the text is talking about and the latter in capturing the red thread, or in other words, ‘what the text is talking about.’^23^

We discussed codes, sub-categories, main categories and themes until consensus was reached. All the identified sub-categories, the main categories, and themes are presented in Table 1.

## Results

The analysis resulted in three themes: To be invited or not, to fit in or not and to contribute or not.  In the first theme, to be invited or not, the participants talked about their specialty choice and described their positive feelings when being invited, feeling valued and trusted. On the other hand, many of the participants expressed an absence of those feelings. They shared stories about positive and negative encounters within the healthcare sector, from their first clinical rotations during medical school to the time when their actual specialty choice was made. Perceived poor working environment contributed to their avoiding certain specialties (and/or individual workplaces). A good working environment, conversely, increased the chances that study participants would consider a future in a particular specialty. Both situations are described from interview excerpts below:

“Unfortunately, it’s definitely the case that, having seen the working conditions at some places, you just turn your back on some options and don’t even consider them…That department and that place seem to be awful places to work. And as they don’t have any competition, they can just carry on like that. You work late several hours every day, and it just seems awful. So, I’m just glad that my interests and specialty aren’t [that way]…I mean, so far in psychiatry, I haven’t had to deal with any of that, in fact, the working conditions have been great.” (No. 9, Psychiatry, woman)

“Yeah, I think that both positive and negative interactions have affected me. On the negative side, I was on a surgical placement there in city-X, and I remember that I had a few negative encounters and experiences while working there, both with the workload, but also with a bit of a lack of support from colleagues, and that made me feel hmm—is this really what I want to be doing? Do I want—do I want this kind of tone, this way of interacting? On the other hand, during medicine I experienced loads of support from my colleagues; you always felt that someone was backing you up and that you could ask questions and get help. That was pretty much the deciding factor.” (No. 3, Internal medicine, man)

Participants also described meetings with, and comments from, superiors, which had a positive effect on their choice of specialty. These positive encounters occurred during clinical rotations when study participants were undergraduate students or graduates, but prior to specialist training. This illustrates the importance of individuals’ influence on young doctors’ career choices. In the following quote, a participant describes the first time she considered becoming an orthopedic surgeon:

“It wasn’t really anything I’d thought about when I started medical school…and then eventually I was doing orthopedics here and met a really enthusiastic orthopod, he’s not here anymore, it was when I was a student. I was—I think I was doing anesthetics and was hanging around in the corridor one evening waiting to do something and he was like, ‘Aha! A medical student—I need an assistant! You’ll need to stick around the whole evening, we’re doing this, this and this. This is going to be revolutionary and new and so on and so on. Yes!’ It turned out to be a really fun evening, and after that I was hooked and ended up meeting a colleague doing some research, so I started doing some work with her, and we had a bit of contact, so—so from then on I pretty much thought, well, yes…if I hadn’t met that person that evening in the corridor, maybe I wouldn’t have fallen in love with orthopedics and maybe not…Because it’s really nothing I’d considered earlier in my medical education, that I would become an orthopod, it wasn’t part of my world.” (No. 1, Orthopaedics, woman)

In the above quote, the senior doctor opened the eyes of the student to the possibility that she had not thought of. Her experiences during that night shift had an impact on her choice. To be noticed and appreciated by someone senior creates a feeling of being valued and needed.

The study participants also stressed the importance of the experience of being trusted. Another participant, training to become a specialist in geriatrics, talked about trust and the importance of feeling confident in having the right kind of support when needed:

“Yes, I remember I met lots of patients with, for example, clinical signs and I thought that was exciting. That, yes, that I got an idea of different illnesses and so on, that I learned things. And I remember the supervisor I had; he gave me a lot of freedom, although still within limits, you know what I mean? I mean he was there the whole time, but he was like “Go and palpate that!” and then he trusted me…I felt like I improved that week—that kind of thing. Yes. Yes. It was informative and enjoyable.” (No. 12, Geriatrics, woman)

**Table 1:** Sub-categories, main categories, and themes

Sub-category	Main category	Theme
The meaning of work environment and superiors’ attitudes towards me	Positive or negative encounters in the healthcare sector before starting specialist training	To be invited or not
Positive or negative perceptions of personality traits	Self-perception in relation to what type of a doctor I think I can be	To fit in or not
How my skills, interests, and perceptions can contribute to my fitting in		
How my personality traits can be beneficial in a particular specialty or some specialist areas	Self-perception in relation to what type of doctor I want to become	To contribute or not
Positive and negative feelings about medical aspects, patient relations and types of work procedures		

Role models play a vital part in the process of choosing a specialty, developing a professional medical identity and, as this resident in surgery put it:

“…the reason to carry on with surgery. There are lots of aspects. One is all the impressive surgeons I’ve met…when I was doing surgery as a medical student, there were several who really made an impression on me. They became my idea of how to be…a doctor, and in some way[s]…an adult.” (No. 6, Surgery, man)

In summary, the first theme showed that working environment—both good and bad—had an impact on our study participants’ specialty choices, and meetings with role models was also crucial for doctors’ choice of specialty.

For the second theme, the participants talked about their own personality and reasoned about how personal traits had influenced them in their specialty choice. Statements about personality had both positive and negative meanings for the participants. The negative connotations were related to a feeling of not having the right kind of personality for some of the specialties. This had nothing to do with competence or skills, but rather with a sense of having a personality that would make it problematic to be a good doctor or to thrive in some of the specialties.

“Gynae and general practice are things that I’ve also thought a lot about. But I felt that they weren’t really good for me as a person, because I get too involved and then I don’t think I’d have the energy. I don’t think that—I mean I think I would have been good at it, I don’t know, now maybe I’m being a bit big-headed, but I don’t think it would have been good for me…And I feel it’s quite important to be-be able to last a lifetime.” (No. 1, Orthopaedics, woman)

Other personality traits had positive connotations. Being calm, accurate, intellectual, curious, practical, good at listening and communicating and easy to cooperate with were all personality traits the participants considered useful in some specialty areas.

“Inquisitive I think, I don’t really know. Curious[ity] to be able to see all the different, you could say, complex problems that a patient [has]…like an illness is a problem, so that the complex problems that a patient has, that with [an] interest[ed] approach [to] all the problems that a patient has, and work with hypotheses and then discard them and make sure you do a thorough workup in a structured way and to maintain an interest and a doggedness in that.” (No. 3, Internal medicine, man)

“You also need to be both thoughtful and decisive in some way, how can I put it? It’s not like you just make quick decisions, you make decisions based on experience and sometimes on fairly loose grounds. I mean there’s no manual of geriatrics like there is for cardiology, for example. I mean there’s very little research about the elderly because they, well, they’re excluded from studies because they have so many illnesses at once, loads of medicines and the likes. So, in some way you have to use many aspects of yourself, reflect and weigh in many factors, for example, ethics is a very big part.” (No 4, Geriatrics, woman)

Another aspect of the theme to fit in or not had to do with a specialty’s inherent characteristics, such as whether the specialty was broad (e.g. primary care) or narrow (e.g. laboratory specialty) and variation in work tasks. Study participants talked about the dichotomy of wanting to work in a broad or a narrow specialty. Within this dichotomy, both aspects were equally important, but whereas some of the participants could only see themselves in a narrow specialty, others could just picture themselves in a broad one.

“It’s about feeling that I can manage a broad area of medicine and that I can actually follow my patients and see what happens to them. It’s really that that attracts me, so I hope to be able to work with that.” (No. 13, Primary care, woman)

Another specialty characteristic brought up by many participants was the wish for variation at work:

“I’ve always thought that I’d like a job with lots of variety, because variety, well, for me that’s the most important factor so I don’t get fed up with the job. That there’s variation, includes more acute elements, so work at the emergency department or critical care in a hospital. Then we’ve got clinics that are non-acute care, so that provides more varied work, and I’ve come to the conclusion that there’s enough variation to stop me [from] getting bored with my job.” (No. 2, Internal medicine, man)

A third aspect of this theme was future career possibilities in the chosen specialty. For some of the participants, the option to work elsewhere in the country or abroad played a part in their choice. For others, it mattered if they would be able to open their clinic or have a variety of employers to choose from. These aspects represent other features of the theme of fitting in.

“And also that there are loads of opportunities to work in other places, both to work near to where you live and that it’s not—that it’s one department in Stockholm and one in Malmö and that’s your only potential employers. It’s an important factor that you can move to a different part of the country if you want.” (No. 13, Primary care, woman)

In summary, the second theme shows that a wish to fit in was a drive for specialty choice. Personality traits, self-perception, and possibilities in the specialty all mattered much more in the complex process of specialty choice.

In the third and last theme, the participants talked about their personal wish to contribute and to choose a specialty where their individual personality, skills, and knowledge could be of use to patients and the healthcare sector. The results in this theme suggest that value related to doing what is best for the patient was related to the study participants’ specialty choice. For some of the study participants, long-term patient relationships were valued with emphasis on relationship building and patient communication. Other study participants emphasize solving medical problems and thought that they could contribute to patients’ wellbeing without long-term relationships.

“A patient comes in, they have a problem, you can do something about it. Not always, but you can often do something about it…in most cases you fix it, so the patient gets better. You see results.” (No. 1, Orthopaedics, woman)

The patients’ medical condition was also central to this theme. Some of the study participants wanted to work with patients that were relatively easy to help; some wanted to work with complex medical problems, while others thought they could contribute most to the care of elderly patients where medical treatment was sometimes no longer an option.

“And in some way, I felt that this was kind of a group of patients [with mental health problems] who weren’t so well looked after. There was a lot you could do. And I felt pretty early on that I had something to contribute. And there’s a lot more complexity that is—that is so difficult that sometimes it can be difficult to do so much about it, really. But you can really help these people, and most of them turn out well if you do the right things. And that’s really satisfying. It’s really rewarding to do that.” (No. 7, Psychiatry, man)

“Because often it’s those who are in such a bad way, it’s often due to, let’s say, natural aging and illness. If you are, for example, 95 years old and maybe getting worse and worse due to pneumonia. It’s our decision to say when enough is enough so to speak. So, things are a bit different for us. And the stress is, on the other hand, about talking to relatives and saying, well, we can’t do too much more. We can’t cheat death. So, for us things are a bit topsy-turvy, because we often decide to give up, we—because we can see that, well, it’s not going anywhere. We’re just prolonging the suffering. Exactly. That’s how it is. Okay, what other advantages do I see. Probably that’s it, what I’ve already mentioned—working with the whole situation, which doesn’t just involve medications. You look at the whole patient. Mobility, what kind of help they have at home, where’s the best place for them. What’s most the most helpful at the moment—taking 30 meds, is that really necessary? Maybe five’s enough. That kind of thing.” (No. 14, geriatrics, man).

The majority of the participants in this study were satisfied with their choice of specialty.  Some of them had, however, considered other specialties but abandoned the idea because they perceived that the working situation in that specialty made it impossible to contribute positively. One of the study participants who wanted to be a primary care specialist changed his mind after his foundation years and chose geriatrics instead:

“After having worked in primary care during my foundation period, I changed my mind, you could say that I found it a bit inhumane, the conditions, I found it hard to be a good doctor when you have so little time. Because you were expected to do quite a lot, all the things you talk about wanting to do you have actually to do, you haven’t got the time to listen to the patient, discuss things, consider other aspects. You can only discuss what they came for and interrupt them if they start to broach other subjects. And things like that you should ask about blood pressure and lifestyle at every visit. There’s no time, so of course it never gets done. Instead you have to deal with what the patient came for and got on to the next person quickly. It felt like either you were a good, sensitive, thorough doctor, which you never had time for, or you were very short and just focused on one thing. There’s the risk for mistakes, but you’ve just got to move on if you’re going to get through everyone you’ve got to see in the working day. It’s that simple. And I felt like I wasn’t going to be able to manage that in the long run.” (No. 14, geriatrics, man)

To summarise, the third theme shows that a desire to contribute to patient recovery was central when choosing a specialty. Important aspects were the patient’s general medical condition and the type of patient relations that a job entailed.

Many of the study participants expressed similar worries about their present and future work situation. Long hours, heavy workloads, lack of administrative support and not enough time for patients were factors that were not described as problems caused by specialty per se but were common to the profession in general.

## Discussion

We took an interpretivist approach^17^ in this qualitative study and, in accordance with that stance, we regarded choice as a process.^18^ Our findings support this view; the data suggest that doctors take many factors into account before they make their specialty choice. The complexity of specialty choice was related to the three themes, presented in this study. This could also be understood in terms of medical doctors weighing up where they think they can fit in, what they think is possible and how they feel that they can contribute to the medical profession. Also, the length of the decision-making process is in itself also an important factor. The process of specialty choice sometimes started as early as medical school, and for most of the participants in this study, the process went on for years. Only a few of the interviewed doctors made an early decision that they kept to. The time aspect should be recognized because it means that providers of specialist training should consider recruitment as an ongoing process starting in medical school.

In this study, we identified three equally important themes for medical doctors when they chose their specialty. The first theme, to be invited or not, summarises how important work environment and the influence of a single superior staff member can be to creating feelings of being welcomed and valued as a young doctor. The second theme, to fit in or not, illustrates the importance of being able to use one’s experience and personality to be part of a community of specialists. The third theme, to contribute or not, encompasses the feelings of wanting to add value to the medical field. A wish to use abilities and personality traits to serve patients and the society best was the overarching theme, then, here.

A majority of the study participants declared that they were very satisfied with their specialty choice, and some even said that they loved their job. However, many of the study participants inhabit a work situation that is too demanding. Lambert and colleagues^28^ concluded that ‘quality of life,’ including aspects such as long hours, heavy demands and workload, was the main factor among British doctors rejecting their initial specialty choice. The work situation for medical doctors, regardless of specialty, needs to be addressed. Otherwise we might face even greater problems recruiting and retaining staff in the healthcare system in the future than we do today. Based on our findings, we suggest that further studies are needed to investigate working conditions in relation to a medical specialty. There are previous research contributions on the work environment for doctors in general.^29^^,^^30^ Fink-Miller^31^ found that there are differences in suicide rates among doctors depending on specialty and that there is a need to look further into the work environment of different medical specialties. Furthermore, there seems to be a lack of studies investigating how doctors perform in different specialties. The doctors in the present study expressed a will to contribute. This theme could be developed into studies about ‘how to do good’ as a doctor in relation to the chosen specialty. This should be investigated both on a personal level—how can doctors in different specialties ‘do good’ for patients—and on a societal level.

### Limitations of the study

This study has some limitations. Firstly, the number of interviewed doctors do not allow us to claim generalisability.^16^ Secondly, all participants were working and training to become specialists in the Stockholm area, meaning perspectives from other parts of the country are lacking.

There is always a risk in using only interviews as data material in terms of different biases. The study participants may have a wish, often unconscious, to present themselves positively and uncomfortable answers may not be given.^20^ We did, however, do our best to create an open and relaxed interview atmosphere.

## Conclusions

Medical doctors take many factors into account during the process of specialty choice. Role models during medical school, work environment, patient relations, and patients’ general medical status are all important factors. The complexity of specialty choice is related to the three themes presented in this study: to be invited or not, to fit in or not and to contribute or not. In addition, the extended length of the decision-making process is a factor that needs to be recognized, as a specialty choice should be understood as a long-term process. The results of this study indicate that recruitment to different medical specialties needs to target the entire chain of medical education. There is, however, also a need to identify critical incidents within the chain of medical education. Further research should focus on those incidents and moments in time that mostly form a decision.

### Conflict of Interest

The authors declare that they have no conflict of interest.
